# Supplementary material for: HIVprotI: an integrated web based platform for prediction and design of HIV proteins inhibitors
Source: J Cheminform. 2018 Mar 9;10:12. doi: 10.1186/s13321-018-0266-y (PMC5845081; doi:10.1186/s13321-018-0266-y)
Supplement: Supplementary file 2 — Additional file 2. Source code of HIVProtI web server. [file 13321_2018_266_MOESM2_ESM.zip › HIVprotI_Source-code/hivprotI/weka-3-6-6/doc/weka/filters/unsupervised/attribute/PropositionalToMultiInstance.html]

PropositionalToMultiInstance


---


|  |  |  |
| --- | --- | --- |
| **Overview** \| **Package** \| **Class** \| **Tree** \| **Deprecated** \| **Index** \| **Help** \| **Weka's home** | |  |
| **PREV CLASS**   **NEXT CLASS** | **FRAMES**    **NO FRAMES**     **All Classes** |  |
| SUMMARY: NESTED \| FIELD \| CONSTR \| METHOD | DETAIL: FIELD \| CONSTR \| METHOD |  |


---


## weka.filters.unsupervised.attribute Class PropositionalToMultiInstance

```
java.lang.Object
  weka.filters.Filter
      weka.filters.unsupervised.attribute.PropositionalToMultiInstance
```

**All Implemented Interfaces:**: java.io.Serializable, CapabilitiesHandler, OptionHandler, RevisionHandler, UnsupervisedFilter

---

``` public class PropositionalToMultiInstance extends Filter implements OptionHandler, UnsupervisedFilter ```

Converts the propositional instance dataset into multi-instance dataset (with relational attribute). When normalize or standardize a multi-instance dataset, a MIToSingleInstance filter can be applied first to convert the multi-instance dataset into propositional instance dataset. After normalization or standardization, may use this PropositionalToMultiInstance filter to convert the data back to multi-instance format.
